# Genetic risks of Alzheimer’s by *APOE* and *MAPT* on cortical morphology in young healthy adults

**DOI:** 10.1093/braincomms/fcad234

**Published:** 2023-09-08

**Authors:** Weijie Huang, Jianmin Zeng, Lina Jia, Dajiang Zhu, John O’Brien, Craig Ritchie, Ni Shu, Li Su

**Affiliations:** State Key Laboratory of Cognitive Neuroscience and Learning, Beijing Normal University, Beijing 100875, China; Department of Neuroscience, Neuroscience Institute, Insigneo Institute for In Silico Medicine, University of Sheffield, Sheffield S10 2HQ, UK; School of Systems Science, Beijing Normal University, Beijing 100875, China; Faculty of Psychology, Sino-Britain Centre for Cognition and Ageing Research, Southwest University, Chongqing 400715, China; Beijing Anding Hospital, Capital Medical University, Beijing 100088, China; Department of Computer Science and Engineering, University of Texas at Arlington, Arlington, TX 76019, USA; Department of Psychiatry, School of Clinical Medicine, University of Cambridge, Cambridge CB2 0SZ, UK; Edinburgh Dementia Prevention and Centre for Clinical Brain Sciences, Edinburgh Medical School, University of Edinburgh, Edinburgh EH4 2XU, UK; Scottish Brain Sciences, Edinburgh EH12 9DQ, UK; State Key Laboratory of Cognitive Neuroscience and Learning, Beijing Normal University, Beijing 100875, China; Department of Neuroscience, Neuroscience Institute, Insigneo Institute for In Silico Medicine, University of Sheffield, Sheffield S10 2HQ, UK; Department of Psychiatry, School of Clinical Medicine, University of Cambridge, Cambridge CB2 0SZ, UK

**Keywords:** genetic risk factor, machine learning, MRI, cortical thickness, cortical curvature

## Abstract

Genetic risk factors such as *APOE* ε4 and *MAPT* (rs242557) A allele are associated with amyloid and tau pathways and grey matter changes at both early and established stages of Alzheimer’s disease, but their effects on cortical morphology in young healthy adults remain unclear. A total of 144 participants aged from 18 to 24 underwent 3T MRI and genotyping for *APOE* and *MAPT* to investigate unique impacts of these genetic risk factors in a cohort without significant comorbid conditions such as metabolic and cardiovascular diseases. We segmented the cerebral cortex into 68 regions and calculated the cortical area, thickness, curvature and folding index for each region. Then, we trained machine learning models to classify *APOE* and *MAPT* genotypes using these morphological features. In addition, we applied a growing hierarchical self-organizing maps algorithm, which clustered the 68 regions into 4 subgroups representing different morphological patterns. Then, we performed general linear model analyses to estimate the interaction between *APOE* and *MAPT* on cortical patterns. We found that the classifiers using all cortical features could accurately classify individuals carrying genetic risks of dementia outperforming each individual feature alone. *APOE* ε4 carriers had a more convoluted and thinner cortex across the cerebral cortex. A similar pattern was found in *MAPT* A allele carriers only in the regions that are vulnerable for early tau pathology. With the clustering analysis, we found a synergetic effect between *APOE* ε4 and *MAPT* A allele, i.e. carriers of both risk factors showed the most deviation of cortical pattern from the typical pattern of that cluster. Genetic risk factors of dementia by *APOE* ε4 and *MAPT* (rs242557) A allele were associated with variations of cortical morphology, which can be observed in young healthy adults more than 30 years before Alzheimer’s pathology is likely to occur and 50 years before dementia symptoms may begin.

## Teaser

Using machine learning, Huang *et al*. uncovered that APOE ε4 and MAPT A allele were associated with a more convoluted and thinner cerebral cortex in young healthy adults. The results indicate that the differences in cortical morphology associated with genetic risk factors of dementia can be observed as early as young adulthood.

## Graphical Abstract

**Graphical abstract:**
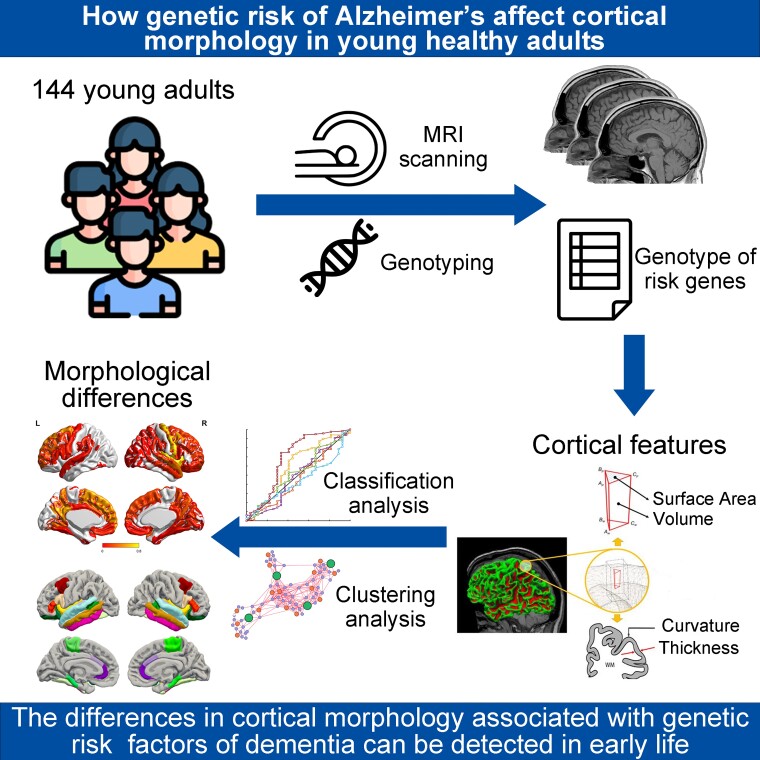

## Introduction

The 2018 Amyloid, Tau and Neurodegeneration (ATN) research framework for Alzheimer’s disease (Ad) provides a systematic method to determine AD continuum designation.^1^ In this framework, extracellular amyloid beta (Aβ) plaques and intracellular hyperphosphorylated tau neurofibrillary tangles are the two most important pathological hallmarks, which precede neurodegeneration. As the most common genetic risk, apolipoprotein E (*APOE*) ε4 relates to impaired Aβ clearance,^2,3^ thus increasing the formation of plaques leading to Ad. Microtubule-associated protein tau (*MAPT*) rs242557 with the major allele G and the minor allele A is also related to tauopathies via differential expression of various exons relevant to tau aggregation.^4‐6^  *MAPT* rs242557 (*MAPT* for short) has been associated with increased risk of Ad, and there is emerging evidence for an additive effect of *APOE* and *MAPT* in modulating the risk for AD.^7^

*APOE* ε4 not only participates during pathological processes in patients with Ad but also impairs cortical structure in non-demented older individuals. For instance, previous studies found that for individuals with subjective cognition decline, *APOE* ε4 carriers showed reduced volume in the left hippocampus^8,9^ and reduced cortical area in the right hemisphere^8,10^ than non-carriers. To the best of our knowledge, there is no existing data about the specific influence of *MAPT* on cerebral structure, but a previous study reported that patients with mild cognitive impairment (MCI) carrying *MAPT* haplotype H1 showed an increased volume in bilateral superior frontal gyri and precentral gyrus as well as in left inferior temporal gyrus and calcarine gyrus.^11^ However, the precise effects of *MAPT* on brain structure and how it interacts with *APOE* ε4 remain unclear.

In addition, a cross-lifespan study showed that genes have a lifelong impact on the trajectory of development and aging of the cortical cortex, in which individuals’ cortical thickness decreased rapidly in the first 20 years of life and after the age of 50 years old but kept relatively stable between 20 and 50 years of age.^12^ Brain areas which showed the most changes before the age of 20 also mirror the areas which showed the fastest decline after the age of 50.^12^ Hence, early 20s is an important time point in life to investigate the early genetic effect on the development of the human cerebral cortex and how genetic predisposition may have a lifelong impact on the brain. However, the existing literature on the effect of *APOE* ε4 on cerebral structure in young adults remains inconsistent. Some studies found that *APOE* ε4 has detrimental effects on hippocampal volume^13^ and entorhinal thickness,^14^ but others found no statistically significant difference between *APOE* ε4 carriers and non-carriers at this stage.^15‐17^ Due to the absence of previous research investigating the relationship between *MAPT* and cerebral structure in young adults, its influence in early life remains unclear. The mixed findings may be attributed to the small sample size in previous studies and the fact that the effect of *APOE* in young adults is too subtle to be detected using conventional univariate analyses. Recent studies highlighted the benefits of multivariable approaches compared with univariate methods when assessing the differences in hippocampal volume between *APOE* ε4 and non-carriers.^18^ In addition, most previous studies only focused on thickness and volume of the cerebral cortex but neglected other measures of cortical folding patterns such as curvature and folding index, which have been demonstrated to be sensitive to Ad pathology.^19^ Finally, most previous studies also suffer from relatively small sample sizes thus the lack of adequate statistical power to detect reliable effects.

Hence, the aim of this study was to use multivariable methods incorporating additional cortical folding information in a large cohort to examine the impacts of *APOE* ε4 and *MAPT* A allele on healthy young adults. We expect ultra-early structural differences to occur in medial prefrontal and temporal lobes, cingulum, precuneus cortex and hippocampus, because these brain regions are known to be vulnerable for early Alzheimer’s pathology.

## Materials and methods

### Participants

A total of 155 self-declared Han Chinese college students aged 18–24 were recruited from Southwest University using advertisements. We have focused on Han population because previous studies showed that the *APOE* ε4 allele is significantly more prevalent in Han than non-Han ethnic Chinese, making Han Chinese at higher risk of developing dementia.^20‐22^ All participants provided written informed consent. This study was approved by the Ethic Committee of Psychological Research at Southwest University, Chongqing City, China. Of the 155 participants, 11 were excluded from the MRI analysis because of excessive head motion.

### Image acquisition and processing

Whole-brain T_1_-weighted scan [MPRAGE, 160 slices, voxel size 1.0 mm,^3^ repetition time (TR) = 2300 ms, echo time (TE) = 2.98 ms, flip angle (FA) = 9°] was acquired using a Siemens Verio 3T MRI scanner. Structural MRI images were processed using FreeSurfer v5.3 (http://surfer.nmr.mgh.harvard.edu/) to calculate cortical area, thickness, curvature, folding index and subcortical volume. Specifically, imaging processing included motion correction and averaging^23^ of multiple volumetric T_1_-weighted images (when more than one is available), removal of non-brain tissue using a hybrid watershed/surface deformation procedure,^24^ automated Talairach transformation, segmentation of the subcortical white matter and deep grey matter volumetric structures (including hippocampus, amygdala, caudate, putamen and ventricles)^25,26^ intensity normalization,^27^ tessellation of the grey/white matter boundary, automated topology correction^28,29^ and surface deformation following intensity gradients to optimally place the grey/white and grey/cerebrospinal fluid borders at the location where the greatest shift in intensity defines the transition to the other tissue class.^30‐32^

Then, the whole-brain MRI images were parcellated into 34 regions per hemisphere according to the Desikan–Killiany Atlas.^33^ Surface area was measured at the grey/white matter boundary, and thickness was measured as the average distances in a region between the white matter and pial surfaces. At each vertex, FreeSurfer measured the mean curvature as follows:


$$H=\;(K_{1}+K_{2})/2,$$

and the folding index as follows:


$$\mathrm{FI}=\;|K_{1}|\times (\left|K_{1}|-|K_{2}\right|),$$

where $K_{1}$ and $K_{2}$ denote the maximum and minimum normal curvature.^34^ Then, the regional mean curvature and folding index were calculated.

### Multivariate discriminative analysis

To construct models that can identify individuals carrying increased genetic risks for Ad, we trained support vector machine (SVM) models and evaluated their performance with 5-fold cross-validation which is a standard method used in machine learning to avoid over-fitting. The pipeline consisted of the following three steps: (i) feature extraction and selection, (ii) model training and evaluation and (iii) identifying the most discriminative features, which we will explain in turn. The below pipeline was performed for classifying *APOE* ε4 positive (*APOE*+) individuals from negative (*APOE*−) individuals and rs242557 A positive (*MAPT*+) individuals from negative (*MAPT*−) individuals, respectively.

#### Feature extraction and selection

For each participant, we extracted cortical area, mean thickness, mean curvature and mean folding index from 68 regions defined by Desikan–Killiany Atlas and volume of the 14 subcortical nuclei as features for SVM. We did not include cortical volume because it is redundant considering that cortical area and mean thickness are included as features. We separated the data set into training and test data set to perform 5-fold cross-validation, and the specific approach to split data is described in the next section. In each fold, we performed generalized linear model (GLM) analyses to compare the differences in all the features between two groups in the training data set while controlling for age, sex and education. For both training and test data sets, only those features with *P*-value <0.05 were kept. Then, we normalized the features with the mean and standard deviations of the corresponding features from the training data set.

#### Model training and evaluation

As we mentioned before, a standard machine learning method with 5-fold cross-validation was used to evaluate the model’s performance. Specifically, we randomly divided the data set into five subsets that have almost the same proportion of participants with higher genetic risk as that of the whole data set with stratified random sampling. In each fold, we used four subsets as training data set and the remaining subset as the test data set. The training data set was used to train a linear SVM model with the soft margin parameter C = 1, and the test data set was then used to evaluate the performance of the trained model. When the 5-fold cross-validation finished, we obtained predicated labels of all participants. The most common measures, accuracy, sensitivity, specificity and the area under curve, were used to evaluate model performance. In addition, we also used positive predictive value and negative predictive value, which depends on not only the model itself but also the prevalence and *F*-score to estimate the models’ performance because these metrics are more reliable in interpreting the classification results from an unbalanced data set. All values are defined as follows:


$$\mathrm{Accuracy}=\frac{\mathrm{TP}+\mathrm{TN}}{\mathrm{TP}+\mathrm{FN}+\mathrm{TN}+\mathrm{FP}}$$

$$\mathrm{Sensitivity}=\frac{\mathrm{TP}}{\mathrm{TP}+\mathrm{FN}}$$

$$\mathrm{Specificity}=\frac{\mathrm{TN}}{\mathrm{TN}+\mathrm{FP}}$$

$$\mathrm{PPV}=\;\frac{\mathrm{TP}}{\mathrm{TP}+\mathrm{FP}}$$

$$\mathrm{NPV}=\;\frac{\mathrm{TN}}{\mathrm{TN}+\mathrm{FN}}$$

$$F=\;2\times \frac{\mathrm{PPV}\times \mathrm{sensitivity}}{\mathrm{PPV}+\mathrm{sensitivity}},$$

where TP, FN, TN, FP, PPV, NPV and *F* denote the number of positive instances correctly predicted (hit), the number of positive instances classified as negative instances (miss), the number of negative instances correctly predicted (correct rejection), the number of negative instances classified as positive instances (false alarm), positive predictive value, negative predictive value and *F*-score, respectively. We then tested each index statistically using permutation testing, which indicates whether the observed index is significantly different from that of random models. Specifically, we re-applied the classification procedure 1000 times. In each run, we permuted all the labels across the samples without replacement. The significance was determined by ranking the above-observed indexes in the null distribution; the *P*-value of each index was the proportion of permutations that showed a higher value than the observed true value.

#### Identifying the most discriminative features

The training of SVM model is based on the determination of the separating hyperplane, which is orthogonal to the discrimination hyperplane or projective direction. It has been shown that the coefficients of the discrimination hyperplane quantify the amount of discriminative feature information.^35,36^ To determine which brain region contributed the most to the prediction, we summed the absolute values of coefficients of all cortical metrics of each region. The absolute values were used to quantify the regions’ contribution to the classification. Similarly, to determine which cortical metrics contributed to the classification, we summed the coefficients of all regions positively related to the outcome and the regions negatively related to the outcome separately.

### Morphological clustering analysis

To investigate the gene–gene interaction between *APOE* and *MAPT* on the imaging features, we used clustering model rather than classification that is unsuitable to evaluate interaction effect. To cluster the regions, we trained a growing hierarchical self-organizing maps (GHSOM) model, which is an artificial neural network and provides a means of representing multidimensional data set as a 2D map.^37^ The features we used here was the same as those in discriminative analysis, and their *Z*-scores were input into model for training. Once trained, this map is a model of the original input data, with individual measures represented as individual weight planes (or layers). Each node corresponds to a weight vector with the same dimensionality as the input data. The nodes in the top layer can be regarded as the centroids of the clusters, i.e. the typical pattern of the class. Then, we assigned every region to the cluster that has the closest distance from the centroids/region. For each participant and each cluster, the intra-cluster distance was calculated by averaging distances from regions to the cluster centroid.

### Statistical analysis

Group comparisons of demographics were performed with analysis of variance and chi-square test. GLM analyses were used to statistically explore the effect of APOE and *MAPT* on cortical morphology characteristics including thickness, area, volume, mean curvature and folding index and subcortical volume controlling for age, sex and education. When comparing volume between groups with different genotypes, total intracranial volume was also controlled for. False discovery rate was used to correct for multiple comparisons in the demographics analysis and univariate analysis.

We used GLM analyses to statistically test for the effects of *APOE*, *MAPT* and their interactions on the four intra-cluster distances controlling for age, sex and years of education. Bonferroni correction was used to correct for multiple comparisons in the clustering analysis. For the cluster showing a significant interaction effect on the intra-cluster distance among groups, we also compared the mean cortical metrics from all regions belonging to that cluster. Threshold for statistical significance was set at *P* < 0.05, two-sided in all analyses.

## Results

### Demographic characteristics of the samples

The samples consisted of 83 individuals without neither genetic risk factor studied here, 11 individuals only carrying *APOE* ε4, 34 individuals with genetic risk due to the *MAPT* locus alone and 16 individuals with both genetic risks. Their age (*P* = 0.784), sex (*P* = 0.393) and education (*P* = 0.536) were not statistically different between groups. Detailed descriptions and the demographics of the samples are provided in Table 1.

**Table 1:** Demographics of all participants

	APOE−		APOE+		*T*/$\chi^{2}$ (*P*)
Gender (M/F)	48/69		11/16		0 (0.978)^a^
Age; yr	18.5–23.9 (20.6 ± 0.9)		19.4–23.4 (20.6 ± 1.0)		0.39 (0.696)^b^
Years of education; yr	12–14 (12.4 ± 0.6)		12–14 (12.3 ± 0.5)		1.21 (0.229)^b^
	MAPT−		MAPT+		*T*/$\chi^{2}$ (*P*)
Gender (M/F)	34/60		25/25		2.58 (0.108)^a^
Age; yr	18.5–23.9 (20.6 ± 0.9)		18.7–23.4 (20.6 ± 0.9)		0.10 (0.921)^b^
Years of education; yr	12–14 (12.4 ± 0.6)		12–14 (12.3 ± 0.6)		0.55 (0.582)^b^
	APOE−MAPT−	APOE+MAPT−	APOE−MAPT+	APOE+MAPT+	*F*/$\chi^{2}$ (*P*)
Gender (M/F)	31/52	3/8	17/17	8/8	2.99 (0.393)^a^
Age; yr	18.5–23.9 (20.6 ± 0.9)	19.5–22.3 (20.4 ± 0.8)	18.7–22.3 (20.6 ± 0.8)	19.4–23.4 (20.7 ± 1.1)	1.07 (0.784)^c^
Years of education; yr	12–14 (12.4 ± 0.6)	12–13 (12.2 ± 0.4)	12–14 (12.4 ± 0.5)	12–14 (12.3 ± 0.6)	2.18 (0.536)^c^

APOE−, APOE ε4 non-carriers; APOE+, APOE ε4 carriers; MAPT−, MAPT rs242557 A non-carriers; MAPT+, MAPT rs242557 A carriers.

a $T/\chi^{2}$ (*P*) value for comparison with a chi-square test.

b $T/\chi^{2}$ (*P*) value for comparison with a two-sample *t*-test.

c $F/\chi^{2}$ (*P*) value for comparison with an analysis of variance.

### Group differences in cerebral structure using univariate analysis

We compared cortical thickness, area, volume, mean curvature and folding index and subcortical volume between *APOE* ε4 carriers and non-carriers using univariate tests. Similarly, we also compared these individual structural characteristics between *MAPT* A carriers and non-carriers. However, no result survived false discovery rate correction.

### Classification between *APOE* ε4 carriers and non-carriers

**Figure 1:**
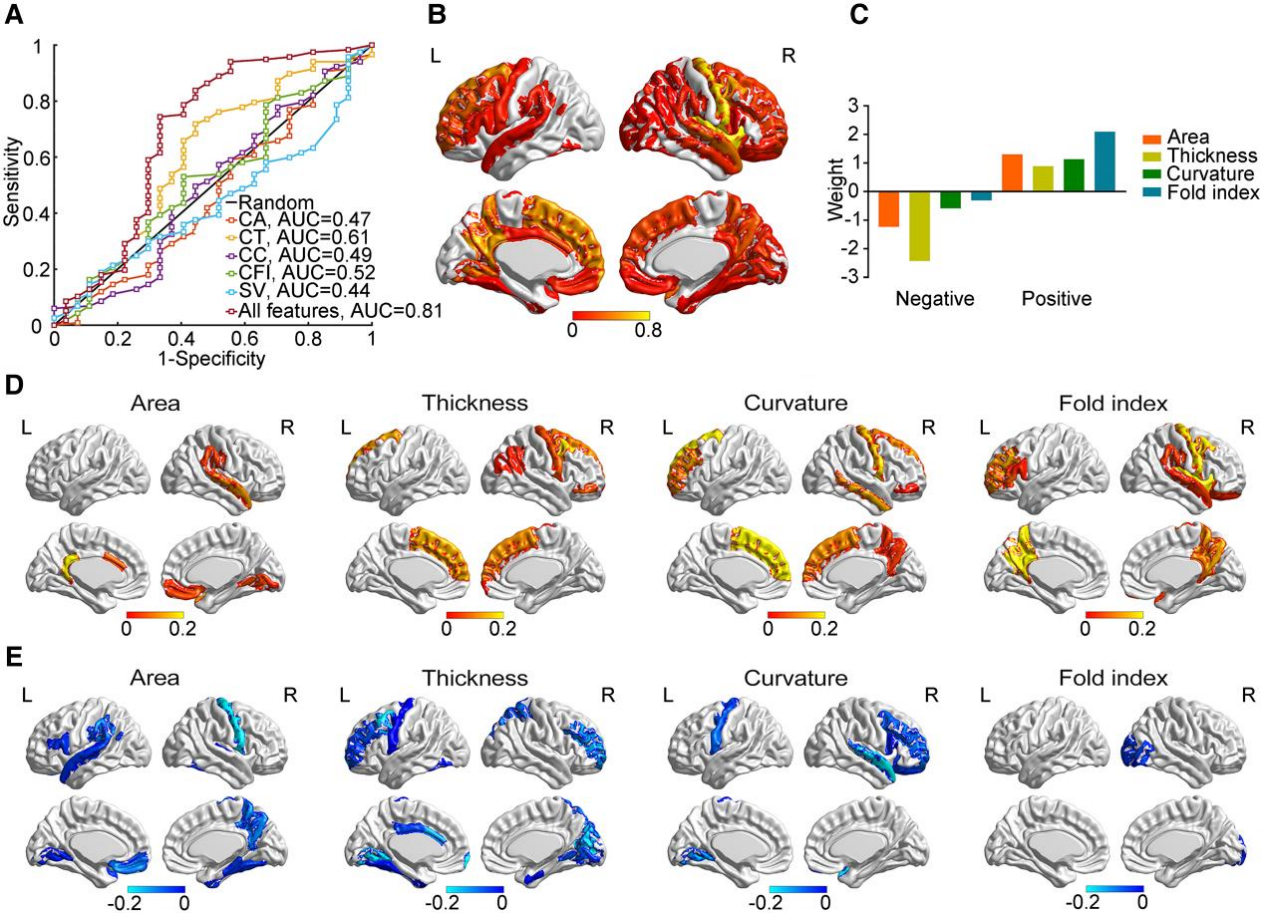
**The classification between *APOE* ε4 carriers and non-carriers.** (**A**) The receiver operating characteristic curve of the classification (*APOE* ε4 carriers, *n* = 27; *APOE* ε4 non-carriers, *n* = 117). (**B**) The map of positive weight. (**C**) The map of negative weight. (**D**) The sum of absolute weight across different measures. (**E**) The sum of weight across different regions. CA, cortical area; CT, cortical thickness; CC, cortical curvature; CFI, cortical fold index; SV, subcortical volume.

We used 5-fold cross-validation to estimate the generalizability of the classifier between *APOE* ε4 carriers and non-carriers. As shown in Table 2, the classifier achieved a classification accuracy of 0.81, a sensitivity of 0.52, a specificity of 0.88 and an area under curve of 0.66. As we mentioned above, the model’s accuracy was also assessed with positive predictive value, negative predictive value and *F*-score that are more robust for our unbalanced data set. These metrices (positive predictive value: 0.50, *P* = 0.011; negative predictive value: 0.89, *P* = 0.001; *F*-score: 0.51, *P* = 0.001) were statistically significantly higher than those of random models. We compared the performance of model using all cortical features and the models using only a single feature. As shown in Fig. 1A, the model using all the features outperformed all other models based on single cortical feature demonstrating the advantage of multivariate approach including multiple cortical features in a single analysis.

**Table 2:** Classification performances

APOE ε4 non-carriers versus APOE ε4 carriers						
	All features	Cortical area	Cortical thickness	Cortical curvature	Cortical folding index	Subcortical volume
Accuracy	**0.81** (0.028)	0.79 (0.086)	0.79 (0.086)	0.81 (0.028)	0.78 (0.217)	0.81 (0.028)
Sensitivity	**0.52** (0.001)	0 (1)	0 (1)	0 (1)	0.07 (0.35)	0 (1)
Specificity	0.88 (0.815)	0.98 (0.038)	0.98 (0.038)	1 (0.001)	0.95 (0.265)	**1** (0.001)
Area under curve	**0.66** (0.016)	0.47 (0.575)	0.61 (0.073)	0.49 (0.435)	0.52 (0.287)	0.44 (0.721)
Positive predictive value	**0.5** (0.011)	0 (1)	0 (1)	0 (1)	0.25 (0.216)	0 (1)
Negative predictive value	**0.89** (0.001)	0.81 (0.506)	0.81 (0.506)	0.81 (0.381)	0.82 (0.272)	0.81 (0.381)
*F*-score	**0.51** (0.001)	0 (1)	0 (1)	0 (1)	0.11 (0.376)	0 (1)

MAPT rs242557 A non-carriers versus MAPT rs242557 A carriers						
	All features	Cortical area	Cortical thickness	Cortical curvature	Cortical folding index	Subcortical volume
Accuracy	0.63 (0.082)	**0.65** (0.013)	0.62 (0.114)	0.63 (0.082)	0.65 (0.013)	0.65 (0.013)
Sensitivity	**0.46** (0.013)	0 (1)	0.12 (0.854)	0.06 (0.964)	0.06 (0.964)	0 (1)
Specificity	0.71 (0.736)	**1** (0.001)	0.83 (0.019)	0.93 (0.005)	0.97 (0.001)	1 (0.001)
Area under curve	**0.61** (0.044)	0.55 (0.168)	0.58 (0.081)	0.48 (0.52)	0.56 (0.152)	0.51 (0.324)
Positive predictive value	**0.47** (0.054)	0 (1)	0.35 (0.402)	0.30 (0.617)	0.50 (0.013)	0 (1)
Negative predictive value	**0.71** (0.022)	0.65 (0.434)	0.65 (0.423)	0.65 (0.476)	0.66 (0.339)	0.65 (0.343)
*F*-score	**0.46** (0.020)	0 (1)	0.18 (0.423)	0.1 (0.963)	0.11 (0.958)	0 (1)

The bold values represent the best performance.

The cortical distribution of positive and negative weights is presented in Fig. 1B and C, respectively, and no subcortical volumes contributed to the classification. Overall, the left superior frontal cortex, left precuneus, left isthmus of cingulate, right precentral cortex, right insula and right caudal middle frontal cortex were the most discriminative regions (Fig. 1D). In addition, the thickness of most brain regions was negatively relative to the presence of *APOE* ε4 and the folding index of most affected brain regions was positively relative to the presence of *APOE* ε4 (Fig. 1E).

### Classification between *MAPT* A carriers and non-carriers

**Figure 2:**
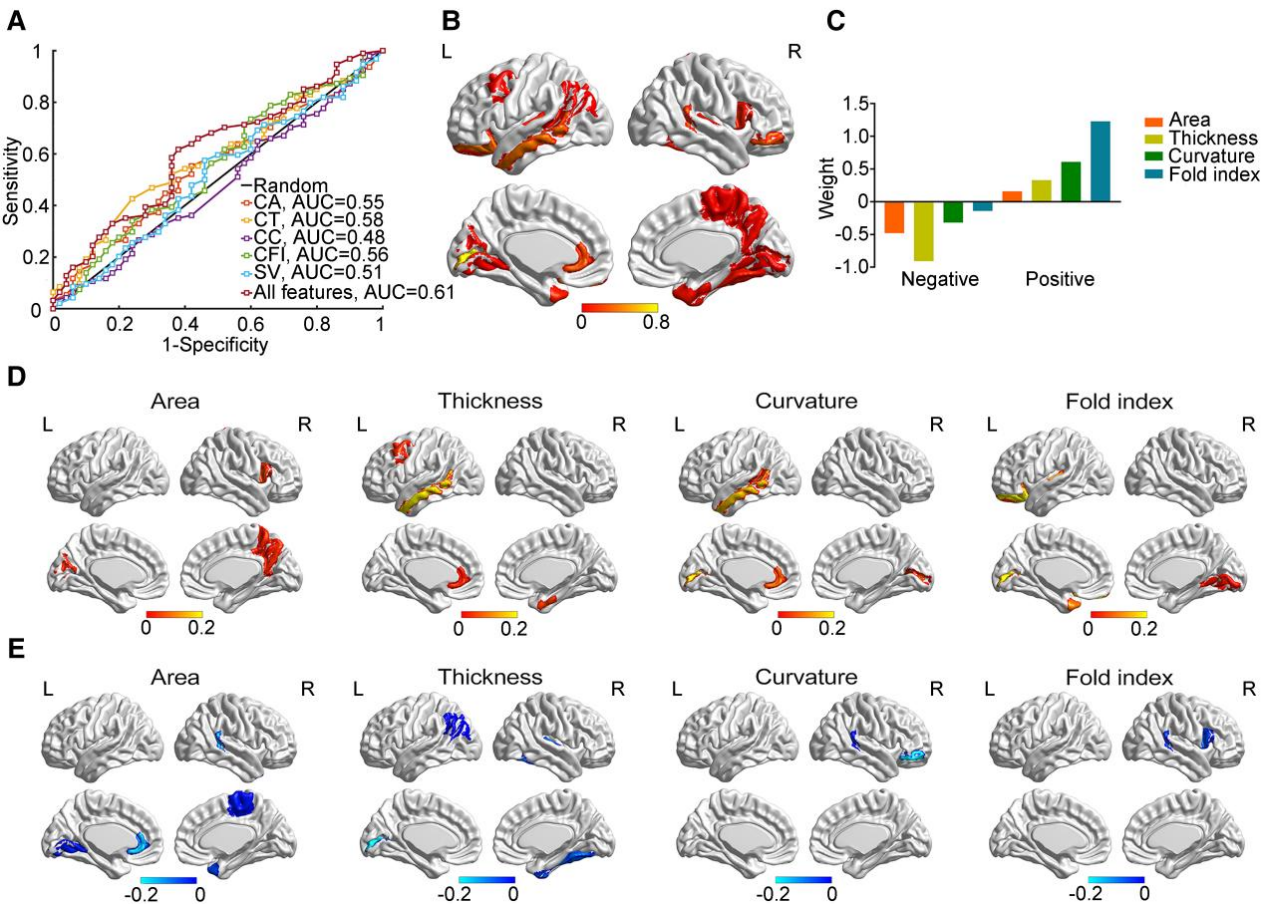
**The classification between *MAPT* rs242557 A carriers and non-carriers.** (**A**) The receiver operating characteristic curve of the classification (*MAPT* A carriers, *n* = 50; *MAPT* A non-carriers, *n* = 94). (**B**) The map of positive weight. (**C**) The map of negative weight. (**D**) The sum of absolute weight across different measures. (**E**) The sum of weight across different regions. CA, cortical area; CT, cortical thickness; CC, cortical curvature; CFI, cortical fold index; SV, subcortical volume.

Similarly, we used 5-fold cross-validation to estimate the generalizability of the classifier between *MAPT* A allele carriers and non-carriers. As shown in Table 2, the classifier achieved a classification accuracy of 0.63, a sensitivity of 0.46, a specificity of 0.71 and an area under curve of 0.61. The positive predictive value, a negative predictive value and an *F*-score of the classifier were 0.47, 0.71 and 0.46, respectively. The last three metrices were more robust and were all statistically significantly higher than that of random models. We compared the performance of model using all features and the models using just a single feature. As shown in Fig. 2A, the model using all features also outperformed other models similar to our previous analysis.

The distribution of positive and negative weights is presented in Fig. 2B and C, respectively, and volume of bilateral hippocampus, bilateral amygdala and right caudate also contributed to the classification. Overall, the left middle temporal cortex, left pericalcarine, left lateral orbitofrontal cortex, rostral anterior cingulate cortex, right pars orbitalis and banks of the superior temporal sulcus were the most affected regions (Fig. 2D). In addition, the thickness of most brain regions was negatively relative to the presence of *MAPT* A allele and the folding index of most affected brain regions was positively relative to the presence of *MAPT* A allele (Fig. 2E).

### *APOE*-*MAPT* interaction on cortical features

**Figure 3:**
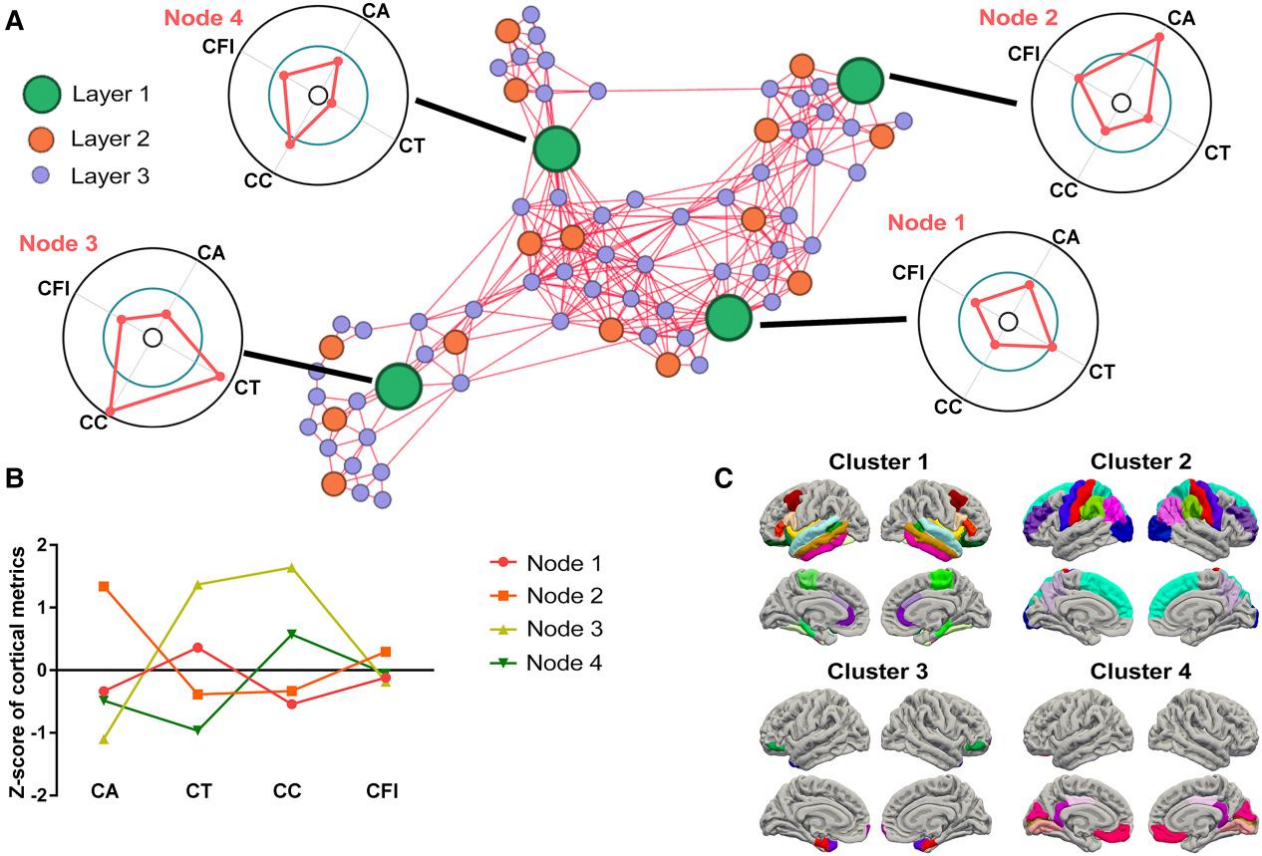
**The cortical morphological profiles defined by neural network and their distributions.** (**A**) Graphical representation of the top three-layer representation, using Euclidean distance and a Force Atlas layout in Gephi toolkit. The biggest nodes correspond to the top layer of nodes, which represent the final clusters, and the radar plots show the profiles of those nodes. (**B**) The profiles of the clusters overlaid. (**C**) The spatial distribution of clusters. CA, cortical area; CT, cortical thickness; CC, cortical curvature; CFI, cortical fold index.

As shown in Fig. 3A, the four nodes in the top layer represent the centroids of four clusters: (i) the brain regions with medium area, large thickness and small curvature; (ii) the brain regions with large area, small thickness and small curvature; (iii) the brain regions with small area, large thickness and large curvature; and (iv) and the brain regions with medium area, small thickness and large curvature. For ease of comparison, the weight profiles of the top layer of nodes are also plotted in Fig. 3B. The spatial distribution of the four clusters across all brain regions is shown in Fig. 3C.

**Figure 4:**
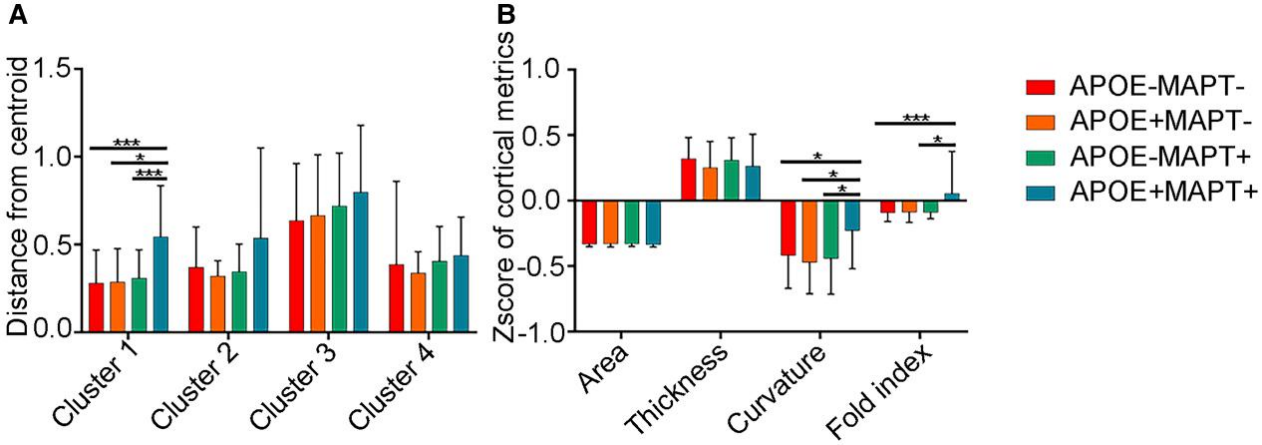
**The interaction effect between *APOE* and *MAPT* rs242557 on cortical morphology.** (**A**) The differences in distance from the centroid of cluster among four groups (*APOE*−*MAPT*−, *n* = 83; *APOE*+*MAPT*−, *n* = 11; *APOE*−*MAPT*+, *n* = 34; *APOE*+*MAPT*+, *n* = 16; interaction between *APOE* and *MAPT P* = 0.009 in GLM). (**B**) The differences in mean cortical metrics of the regions belonging to Cluster 1 among four groups (*APOE*−*MAPT*−, *n* = 83; *APOE*+*MAPT*−, *n* = 11; *APOE*−*MAPT*+, *n* = 34; *APOE*+*MAPT*+, *n* = 16; curvature, interaction between *APOE* and *MAPT P* = 0.019 in GLM; folding index, interaction between *APOE* and *MPAT P* = 0.011 in GLM). *P < 0.05, **P < 0.01, ***P < 0.001. Error bars represent standard deviation. *APOE*−*MAPT*−, participants carrying neither *APOE* ε4 nor *MAPT* rs242557 A. *APOE*+*MAPT*−, participants carrying *APOE* ε4. *APOE*+*MAPT*−, participants carrying *MAPT* rs242557 A. *APOE*+*MAPT*+, participants carrying both *APOE* ε4 and *MAPT* rs242557 A.

**Figure 5:**
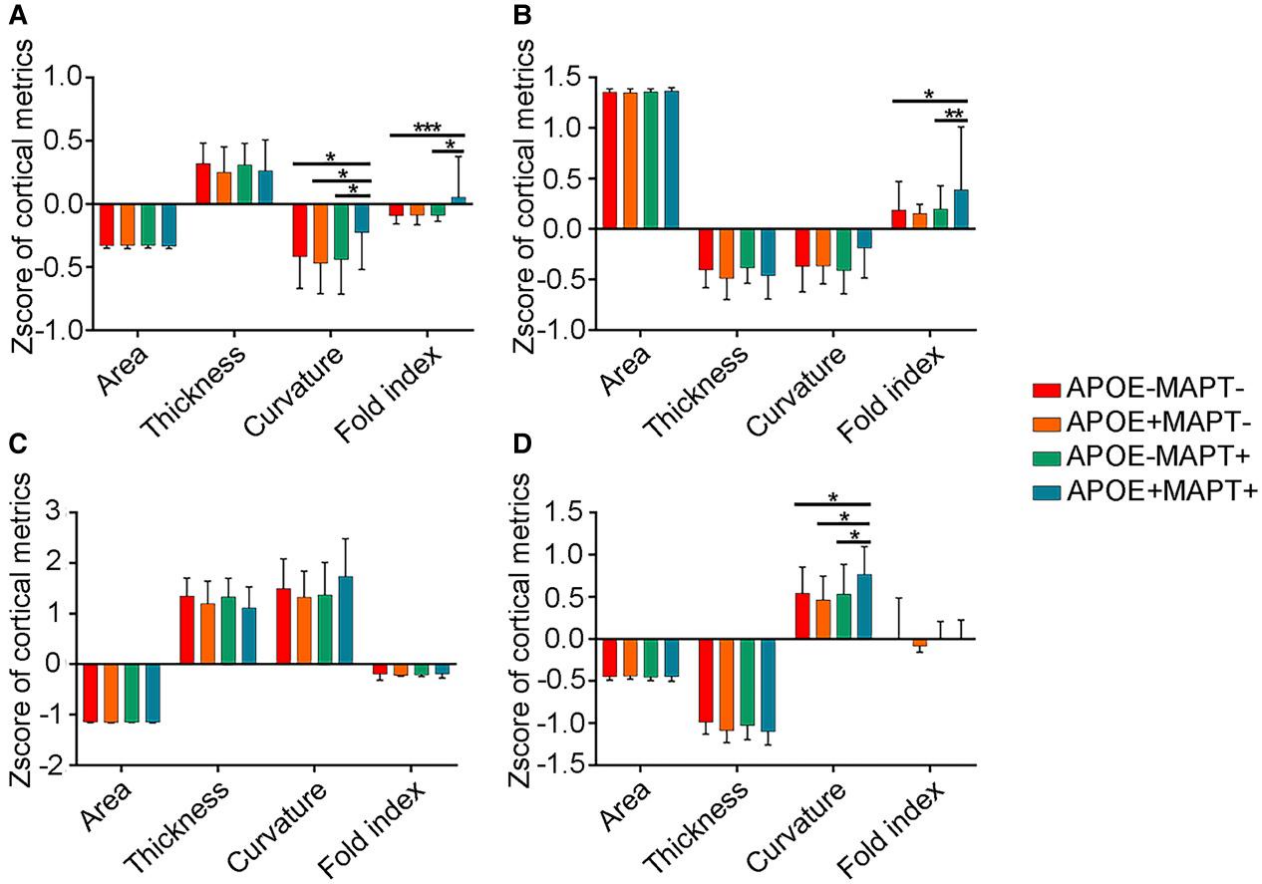
**The interaction effect between *APOE* and *MAPT* rs242557 on mean cortical metrics of four clusters.** (**A**) The differences in mean cortical metrics of the regions belonging to Cluster 1 (*APOE*−*MAPT*−, *n* = 83; *APOE*+*MAPT*−, *n* = 11; *APOE*−*MAPT*+, *n* = 34; *APOE*+*MAPT*+, *n* = 16; curvature, interaction between *APOE* and *MAPT P* = 0.019 in GLM; folding index, interaction between *APOE* and *MPAT P* = 0.011 in GLM). (**B**) The differences in mean cortical metrics of the regions belonging to Cluster 2 (*APOE*−*MAPT*−, *n* = 83; *APOE*+*MAPT*−, *n* = 11; *APOE*−*MAPT*+, *n* = 34; *APOE*+*MAPT*+, *n* = 16; folding index, interaction between *APOE* and *MPAT P* = 0.044 in GLM). (**C**) The differences in mean cortical metrics of the regions belonging to Cluster 3. (**D**) The differences in mean cortical metrics of the regions belonging to Cluster 4 (*APOE*−*MAPT*−, *n* = 83; *APOE*+*MAPT*−, *n* = 11; *APOE*−*MAPT*+, *n* = 34; *APOE*+*MAPT*+, *n* = 16; curvature, interaction between *APOE* and *MAPT P* = 0.026 in GLM). *P < 0.05, **P < 0.01, ***P < 0.001. Error bars represent standard deviation. *APOE*−*MAPT*−, participants carrying neither *APOE* ε4 nor *MAPT* rs242557 A. *APOE*+*MAPT*−, participants carrying *APOE* ε4. *APOE*+*MAPT*−, participants carrying *MAPT* rs242557 A. *APOE*+*MAPT*+, participants carrying both *APOE* ε4 and *MAPT* rs242557 A.

GLM analyses showed a significant interaction (*T* = −2.600, *P* = 0.009) between *APOE* and *MAPT* such that individuals with both genetic risks had the significantly increased distance from the centroid of the first cluster comparing with the other groups (Fig. 4A). This deviation was mainly caused by the increase in curvature (*T* = −2.379, *P* = 0.019) and folding index (*T* = −2.567, *P* = 0.011) (Fig. 4B). While there was no difference in distance from the centroid in other three clusters, we also compared their mean cortical measurements. Similar gene–gene interaction on the folding index in regions from the second cluster (*T* = −2.034, *P* = 0.044) (Fig. 5B) and the curvature of the regions in the fourth cluster (*T* = −2.258, *P* = 0.026) (Fig. 5D) was found.

## Discussion

The present study yielded a main finding that *APOE* ε4 and *MAPT* A allele had a synergetic effect on cerebral cortical morphology especially in a network of brain regions including the bilateral lateral and medial temporal cortex, anterior cingulate, paracentral cortex, insula, lateral orbitofrontal cortex, pars orbitalis and caudal middle frontal cortex as early as early adulthood. The carriers with both risk genes showed a different cortical morphology, namely increasing curvature and folding index and decreased thickness in these regions.

### Early effect of *APOE* on cortical morphology

The SVM model separating *APOE* status based on cerebral structural features showed a significant discriminability between *APOE* ε4 carriers and non-carriers, which indicated that young healthy adults carrying *APOE* ε4 had a different pattern of cerebral structure from non-carriers. By analysing the model’s weights, the individuals carrying *APOE* ε4 have higher folding index and curvature and lower thickness across widespread regions of the cerebral cortex. Regarding the most discriminative regions, the right precentral cortex, right insula, left superior frontal cortex, right superior temporal cortex and right caudal middle frontal cortex were the top five regions that contributed most to the classification. This is consistent with previous studies showing that the superior temporal cortex^11^ and insula^38^ have reduced grey matter volume in amnestic mild cognitive impairment with *APOE* ε4 compared with non-carriers. Furthermore, a longitudinal study showed that *APOE* ε4 accelerated brain atrophy in superior temporal gyrus in healthy older participants.^39^ The right precentral cortex, left superior frontal cortex and right caudal middle frontal cortex showed thinner cortex or lower grey matter volume already in middle-aged healthy individuals with *APOE* ε4.^40,41^ Our results extend the existing knowledge about the relationship between *APOE* ε4 and altered cortical morphology from middle-aged and older people to young healthy adults, when Ad pathology was unlikely.

### Early effect of *MAPT* on cortical morphology

Although the model discriminating the *MAPT* status had a slightly lower performance than the model classifying the *APOE* status, it significantly outperformed random models in terms of the *F*-score test. Across the cerebral cortex, the model for *MAPT* showed a similar pattern as the *APOE* analysis, that is, individuals carrying *MAPT* A allele have higher folding index and lower thickness. The difference between two models is that the number of regions contributing to classifying *MAPT* status is less than that for *APOE*. However, most of these regions identified in the SVM analysis were those implicated in the early phase of Braak pathological staging for tau and amyloid beta, such as the entorhinal cortex, fusiform, lingual, middle temporal, cingulate and temporal pole.^42^ Although we did not have biomarker evidence for tau and amyloid pathology in this study, it is reasonable to assume that our young healthy cohort was free from Ad pathology as well as other comorbidities found in patients with established Ad. So, the current findings might represent a pure genetically determined neuroanatomical trait which makes these individuals more vulnerable to Ad pathology in the future.

### Interaction between *APOE* and *MAPT* in early adulthood

The GHSOM clustering analysis showed that in our cohort, the cerebral cortex could be separated into four major morphologically distinct networks; each is composed of several morphologically similar regions: (i) larger but thinner and less convoluted regions, (ii) smaller and thicker but more convoluted regions, (iii) medium sized and thicker but less convoluted regions and (iv) medium sized and thinner but more convoluted regions. The various morphological pattern might be contributed to large-scale and regionally heterogeneous development in thickness, area and convolution during adolescence and early adulthood.^43‐48^ As expected, the spatial patterns identified in this study are consistent with previous studies.^49‐51^

Further analysis based on these clusters/networks revealed that the individuals carrying both *APOE* ε4 and *MAPT* A had a significant deviation from the typical morphology in the medium sized and thicker but less convoluted brain regions by increasing curvature and folding index. These regions overlapped with the regions discriminating *APOE* and *MAPT* status in the SVM analysis, such as the superior, middle and medial temporal cortex and insular and caudal middle frontal cortex. The supervised classification and unsupervised clustering analysis gave convergent evidence supporting individuals with *APOE* ε4 and *MAPT* A tend to have higher curvature and folding index in these key brain regions. It is worth noting that these regions showed similar development and aging-related cortical thickness change and had a shared genetic influence.^12^ Not only individuals with both genetic risk but also patients with schizophrenia,^52^ 22q11.2 deletions,^53^ autistic and Asperger disorders^54^ and Williams syndrome^55^ showed the similar morphological characteristics, which may suggest that this kind of cortical morphology may be adverse for brain and mental health and increases vulnerability due to Ad pathology in older age.

The process of forming cortical folding during brain development is complex and has not been comprehensively studied yet. However, there are several widely investigated hypotheses arguing that cortical folding is caused by the growth processes during cortical development and/or the mechanical tension within axons.^56^ Based on the axonal hypotheses, it has been speculated that synaptic pruning and reduction of connectivity during normal neurodevelopment may lead to the folding reduction from about 75th week of gestation to almost 23 years of old.^57^ So, genetic risks of Ad may interfere with such pruning process leading to higher folding. Recent studies have also suggested prion-like mechanisms of propagation of Aβ and tau proteins in AD,^58^ so the individual carrying higher risk of Ad may have remaining unpruned connections thus more convoluted cortex. Hyperconnectivity may promote the spread of neurodegenerative pathology when it initiates later in life. Combining with existing knowledge about the effect of *APOE* ε4 and *MAPT* A on Ad, these two genes may already play a significant role in shaping the brain across the lifespan by changing the cerebral structural to be more vulnerable to molecular pathology in the future.

### Limitations

When considering the present study, there are several limitations. First, despite using multiple metrics to evaluate the performance of classification models, the performance of the model might be affected by the unbalance of risk groups.^59^ Although statistically significant, the discriminability of models, especially the model classifying *MAPT*, was modest, so the results await future validation and replication based on larger cohorts. In addition, although our ratios of *APOE* and *MAPT* carriers are consistent with the natural frequency of these genes in non-selected population, the unbalanced groups defined by genetic risks may also impede the generalization of our conclusions to other data sets where genetic risks are enriched by including patients with dementia. Second, the cross-sectional nature of our current study makes it difficult to interpret the results and make causal inference. Although this represents a limitation of our study, it also encourages the development of more comprehensive longitudinal studies to be conducted in the healthy population for brain health related to Ad in the future. Third, all the analyses in this study were based on regions of interest. Considering that the cerebrally structural variation in young adults is subtle, vertex-wised analyses may be more flexible to discover these subtle differences and unbiased by the selection of cortical parcellation. However, the high-dimensionality of imaging data and multiple comparisons problem in vertex-wised analyses introduce computational and other methodological challenges. Finally, future research is needed to consider diversity and inclusivity to cover other non-Han Chinese ethnic minorities because they have both unique and shared genetic and environmental risks for Ad.

## Conclusion

In summary, we discovered that Ad-related risk genes *APOE* ε4 and *MAPT* A have effects on the morphology of the cerebral cortex in healthy young adults ∼40–50 years before Ad symptoms may occur. Our results suggest that such risks may be reflected by altered cortical morphology already detectable in healthy young adults. In addition, this study demonstrated that more attention should be paid to combine multivariate cortical features such as curvature, folding index and area in future studies and young healthy adults are at an important stage in which Ad risk genes start to have an impact.

## Data Availability

Anonymized data can be made available upon request if provided with aims and analysis plan. The authors will review the requests and decide whether data sharing is appropriate based on scientific rigour of the proposal and regulations on personal data protection at the time.
